# Lymphocytes Are Not Required for Neurogenic Heterotopic Ossification Development after Spinal Cord Injury

**DOI:** 10.1089/neur.2021.0072

**Published:** 2022-02-22

**Authors:** Kylie A. Alexander, Hsu-Wen Tseng, Irina Kulina, Whitney Fleming, Cedryck Vaquette, François Genêt, Marc J. Ruitenberg, Jean-Pierre Lévesque

**Affiliations:** ^1^ Mater Research Institute, The University of Queensland, Translational Research Institute, Woolloongabba, Queensland, Australia.; ^2^ School of Dentistry, The University of Queensland, Herston, QLD, Australia.; ^3^ UPOH (Unité Péri Opératoire du Handicap, Perioperative Disability Unit), Physical and Rehabilitation Medicine department, Raymond-Poincaré Hospital, Assistance Publique–Hôpitaux de Paris (AP-HP), Garches, France.; ^4^ Versailles Saint-Quentin-en-Yvelines University (UVSQ); UFR Simone Veil—Santé, END: ICAP, Inserm U1179, Montigny-le-Bretonneux, France.; ^5^ Garches Neuro-Orthopaedics Research Group (GRENOG), Garches, France.

**Keywords:** B lymphocytes, bone, neurogenic heterotopic ossification, spinal cord injury, T lymphocytes

## Abstract

Neurogenic heterotopic ossifications (NHOs) are incapacitating complications of traumatic brain and spinal cord injuries (SCI) that manifest as abnormal bone formation in periarticular muscles. Using a unique model of NHO after SCI in genetically unmodified mice, we have previously established that the innate immune system plays a key driving role in NHO pathogenesis. The role of adaptive immune cells in NHO pathogenesis, however, remains unexplored in this model. Here we established that B lymphocytes were reduced in the spleen and blood after SCI and increased in muscles of mice in which NHO develops, whereas minimal changes in T cell frequencies were noted. Interestingly, *Rag1*^-/-^ mice lacking mature T and B lymphocytes, developed NHO, similar to wild-type mice. Finally, mice that underwent splenectomy before SCI and muscle damage also developed NHO to the same extent as non-splenectomized SCI controls. Overall, our findings show that functional T and B lymphocytes have minimal influence or dispensable contributions to NHO development after experimental SCI in mice.

## Introduction

Neurogenic heterotopic ossifications (NHOs) are abnormal formations of extraskeletal bones in periarticular muscles^1^ that can develop after damage to the central nervous system (CNS), including spinal cord injury (SCI), traumatic brain injury (TBI), stroke, and cerebral anoxia.^2^ NHOs develop in 15–25% of patients with SCI and 5–12% of those with TBI,^3,4^ with the highest prevalence (>60%) seen in combat-inflicted traumas, particularly in victims of explosive blasts with associated SCI or TBI.^5,6^ NHOs are debilitating because of their size (up to 2 kg), causing pain and reduction in the range of motion in affected limbs, often progressing to complete joint ankylosis. This exacerbates functional disabilities by increasing difficulty in sitting, eating, and dressing.^7^ NHOs also cause nerve and blood vessel compression, and can irreversibly damage the affected joints, further increasing patient morbidity.^8^

Management is limited to surgical resection once NHOs are diagnosed and have become symptomatic.^2,7,9–11^ The development of alternative approaches to manage NHO has been slow, and trials of pharmacological interventions continue to show limited effectiveness, reflecting the current limited knowledge on the etiology and pathophysiology of NHO.

We previously developed the first clinically relevant model of SCI-NHO in genetically unmodified mice, where NHO development requires the combination of SCI and a muscle injury induced by the intramuscular injection of cardiotoxin.^12^ In this model, mineralized calcium deposition is detected by micro-computed tomography (μCT) as early as day 3 post-SCI and muscle injury.^12^ NHO bone volumes peak around day 7 to 14, where osterix-positive osteoblasts lay down collagen I and osteocalcin-containing bone matrix on NHO surfaces, which results in the development of small heterotopic bone nodules via intramembranous ossification.^13–15^ These bone nodules subsequently undergo remodeling via multi-nucleated osteoclasts between weeks 2 and 3 post-injury.

Using this model, we have already established that cells of the innate immune system play vital roles in NHO pathogenesis.^12^ Specifically, we have demonstrated that SCI exacerbates and prolongs macrophage-mediated inflammatory responses in injured muscles, and that *in vivo* depletion of macrophages prevents NHO development.^12^ The protracted macrophage-mediated inflammation observed within injured muscles of SCI mice coincides with an accumulation of the proinflammatory cytokine oncostatin M^16^ and overstimulation of the Janus kinase (JAK)1/2-signal transducer and activator of transcription (STAT)3 pathway downstream of the oncostatin M receptor. Administration of a JAK1/JAK2 tyrosine kinase inhibitor ruxolitinib significantly attenuated NHO development.^13^ We have also established redundant roles for neutrophils, which, too, can produce oncostatin M^17^; however, NHO developed in neutropenic mice, similar to control mice.^18^

While the contributions of different innate immune cells are now well established in both genetic^19,20^ and trauma-induced HO,^21,22^ possible roles for adaptive immune cells remain less well characterized and/or understood. Lymphocytes are present at early stages of fibrodysplasia ossificans progressiva (FOP), a fully penetrant and lethal genetic form of HO,^23,24^ while mice lacking functional T and B lymphocytes (*Rag1*^-/-^ mutant mice) have been shown to develop less HO after non-neurological trauma.^25^

Possible involvement of lymphocytes in HO development and/or progression is further supported by the observation that interleukin-3 (IL-3), a cytokine produced by activated T lymphocytes, is associated with HO development after combat-associated trauma in humans.^26^ Conversely, however, pre-operative hip irradiation before hip arthroplasty, a treatment proposed for the prevention of HO in humans, was shown to elevate CD8^+^ T cells, and this was hypothesized to help prevent HO formation.^27^

Dual and seemingly opposing roles for adaptive immune cells in bone dynamics have been described where they influence bone formation,^28^ resorption,^29–31^ and disease.^32,33^ Interestingly, fracture healing was also reported to be either accelerated^34^ or delayed^35^ in *Rag1*^-/-^ mice,^36^ while B lymphocytes were shown to play a minimal role in intramembranous bone formation during bone healing.^37^

Last, traumatic SCI—a condition that predisposes to NHO development—is often hallmarked by acute lymphopenia in both humans and mice.^38–40^ In view of these reports, we investigated whether B and T lymphocytes play a role in SCI-induced NHO pathogenesis in our mouse model.

## Methods

### Animals

C57BL/6 and *Rag1*^-/-^ mice (B6.SVJ129-*Rag1*^tm1Bal/Arc^; backcrossed >10 times into C57BL/6 genetic background) were sourced from the Animal Resource Center (Perth, Australia). *Rag1*^-/-^ mice contain a homozygous inactivating mutation of the recombinase recombination activating 1 (*Rag1*) gene that prevents V(D)J recombination in the T and B cell receptor as well as immunoglobulin genes, thereby blocking maturation of B and T lymphocytes at the pre-pro B and double negative thymocyte stage, respectively. As a consequence, *Rag1*^-/-^ mice lack mature T and B lymphocytes.^37^

All mice were housed at the Translational Research Institute, Biological Research Facility (Queensland, Australia) under specific pathogen-free conditions and fed a standard diet chow (Specialty Feeds, Western Australia, Australia) with *ad libitum* water access and simulated diurnal cycle. Mice were randomly assigned into cages with a maximum of five mice per cage. Mice with different genotypes, all at 5–6 weeks of age, were cohoused in cages containing mixed animal cohorts (for example: 2–3 C57BL/6 with 2–3 *Rag1*^-/-^ mice) 5–7 days before surgery. All mouse procedures were approved by the Health Sciences Animal Ethics Committee of The University of Queensland (ethics numbers 2014/AE000054 and 2017/AE000050) and performed in accordance with the Australian Code of Practice for the Care and Use of Animals for Scientific Purposes.

### NHO mouse model

The NHO mouse model was performed as described previously.^12^ Under general anesthesia (100 mg/kg ketamine, 10 mg/kg xylazine, and 1% isoflurane), 6–7 week old female mice received a laminectomy on the dorsal spine, and the spinal cord was transected between thoracic vertebrae T11 and T13. Mice subsequently received either an intramuscular injection (im) of cardiotoxin (CDTX; 0.32 mg/kg body weight in a volume of 4.7 μL/g body weight), purified from the venom of *Naja pallida* snake (Latoxan, Portes les Valence, France), or phosphate-buffered saline (PBS) into the hindlimb hamstring muscle as described previously.^2^

Post-surgery, all mice received a subcutaneous injection of ciprofloxacin (10 mg/kg) as prophylactic antibiotherapy and buprenorphine (0.05 mg/kg) for pain relief. As SCI causes paraplegia, bladders were manually voided twice daily, and mice were given Bactrim (800mg/L, Roche) in their drinking water as a prophylaxis to prevent urinary tract infections.

### Splenectomy and sham surgery

Splenectomy was performed as described previously.^41^ Briefly, mice were anesthetized, and fur was sprayed with 70% ethanol. A small incision through the skin and abdominal muscle layers was performed to open the peritoneal cavity and enable manipulation of the spleen. Splenic blood supply was cut off by ligating blood vessel bundles twice with silk thread, following which the spleen was removed from the peritoneal cavity and the surgical site closed with sutures. For Sham surgery, the peritoneal cavity was opened to expose the spleen; however, sutures were not placed around the splenic vasculature, and the spleen was not removed. Splenectomies were performed immediately before SCI surgery and CDTX injection as described above.

### Tissue collection

For collection of the spleen for flow cytometry, the whole spleen was dissociated in a total of 10 mL PBS containing 2% fetal calf serum (FCS) using a GentleMACS Dissociator tissue homogenizer with matching C tubes (Miltenyi Biotec, Macquarie Park, Australia) on “spleen 3” setting, twice. For collection of muscle for flow cytometry, hamstrings were cut into 1 mm pieces, and up to 0.5 g of tissue was used per skeletal muscle dissociation kit as per manufacturer's instructions (Miltenyi Biotech, Germany). Bone marrow was isolated by flushing one femur with 1 mL PBS containing 2% FCS. Then, 0.5 to 1.0 mL of blood erythrocytes were harvested by terminal cardiac puncture under anesthesia, and blood erythrocytes were lysed as described.^42^

### Flow cytometry

Total leukocyte number/populations in muscle, blood, and spleen were identified into multiple populations using a Beckman Coulter Life Sciences CytoFLEX benchtop flow cytometer using the following antibodies (Biolegend): fluorescein isothiocyanate (FITC) or phycoerythrin (PE) anti-mouse CD3ɛ (clone 145-2C11), Brilliant Violet 785™ or allophycocyanin (APC)/Cyanine7 anti-mouse CD45 (clone 30-F11), Pacific Blue™ or Brilliant Violet 510™ anti-mouse/human CD11b (clone M1/70), APC/Cyanine7 or biotinylated anti-mouse/human CD45R/B220 (clone RA3-6B2), Brilliant Violet 510™ anti-mouse/human CD11b (clone M1/70), APC/Cyanine7 or PE/cyanine7 anti-mouse CD8a (clone 53-6.7), Peridinin Chlorophyll (PerCP)/Cyanine 5.5 anti-mouse Ly-6G/Ly-6C (Gr-1) (clone RB6-8C5), APC anti-mouse F4/80 (clone BM8), Pacific Blue™ anti-mouse CD4 (clone GK1.5), Pacific Blue™ anti-mouse Ly-6C (clone HK1.4), and streptavidin A700, or from BD Biosciences: PE anti-mouse NK-1.1 (clone PK136).

Live/dead discrimination was performed using either BD Horizon™ Fixable Viability Stain 700 (BD Biosciences) or 7-aminoactinomycin D (Life Technologies). Data files were analyzed with Flow Jo software version 10.7.

### μCT and NHO volume quantification

Because of instrument upgrades during the course of this study, NHO volumes were measured either in vivo or *ex vivo* using either the Inveon positron emission tomography/computed tomography (PET-CT) multi-modality system (Siemens Medical Solutions Inc.), the Molecubes β-Cube and X-Cube μPET-CT system (Molecubes), or the μCT40 scanner (SCANCO Medical AG, Brüttisellen, Switzerland).

For samples measured using the Inveon, parameters were: 360 degree rotation, 180 projections, 500 msec exposure time, 80 kV voltage, 500 μA current, and an effective pixel size of 36 μm. The three-dimensional (3D) reconstitutions were performed using the Inveon Research Workplace software (Siemens Medical Solutions Inc). To quantify NHO volumes, the region of interest (ROI) was drawn around the muscles containing NHO, and these were then carefully checked from three dimensions to ensure adjacent long bones were not included in the ROI. Calcified NHO regions were defined as above the threshold of 450 Hounsfield units (HU).^13,16,18^

Sample analysis using the μCT40 scanner were performed at a resolution of 30 μm, and 3D images of the lower parts of the mouse bodies were reconstructed from the scans using the μCT system software package. Quantitative assessments of bone volumes to detect and quantify NHO were performed using a subtraction technique of orthotopic mouse skeleton (hip, femur, tibia, fibula).^12^ Scanner settings for the X-Cube unit (Molecubes, Belgium) were: 50kV X-ray voltage, 350 μA current, 960 exposures at 32 msec each, continuous single rotation, and binning factor of 1. Images were reconstructed at 50 μm isotropic voxel size using the Feldkamp algorithm while bone analysis was performed using PMOD software v4.1.2 (PMOD Technologies, Switzerland) and Inveon Research Workplace v4.2 (Siemens, Germany).

### Statistical analysis

Data are presented as mean ± standard deviation. Statistically significant differences were determined using the two-sided Mann-Whitney test or one-way analysis of variance (ANOVA) with multiple comparisons in PRISM 8 (GraphPad software, La Jolla, CA).

## Results

### Lymphocytic cells are present within muscles in which SCI-NHO develops

We first established the relative frequencies of B and T lymphocytes across multiple tissue compartments in our SCI-NHO mouse model. Leukocytes were isolated from muscle, blood, and spleen at four days post-surgery, a time point we have established previously to be the peak of innate immune cell infiltration in injured muscles.^13^

**FIG. 1.**
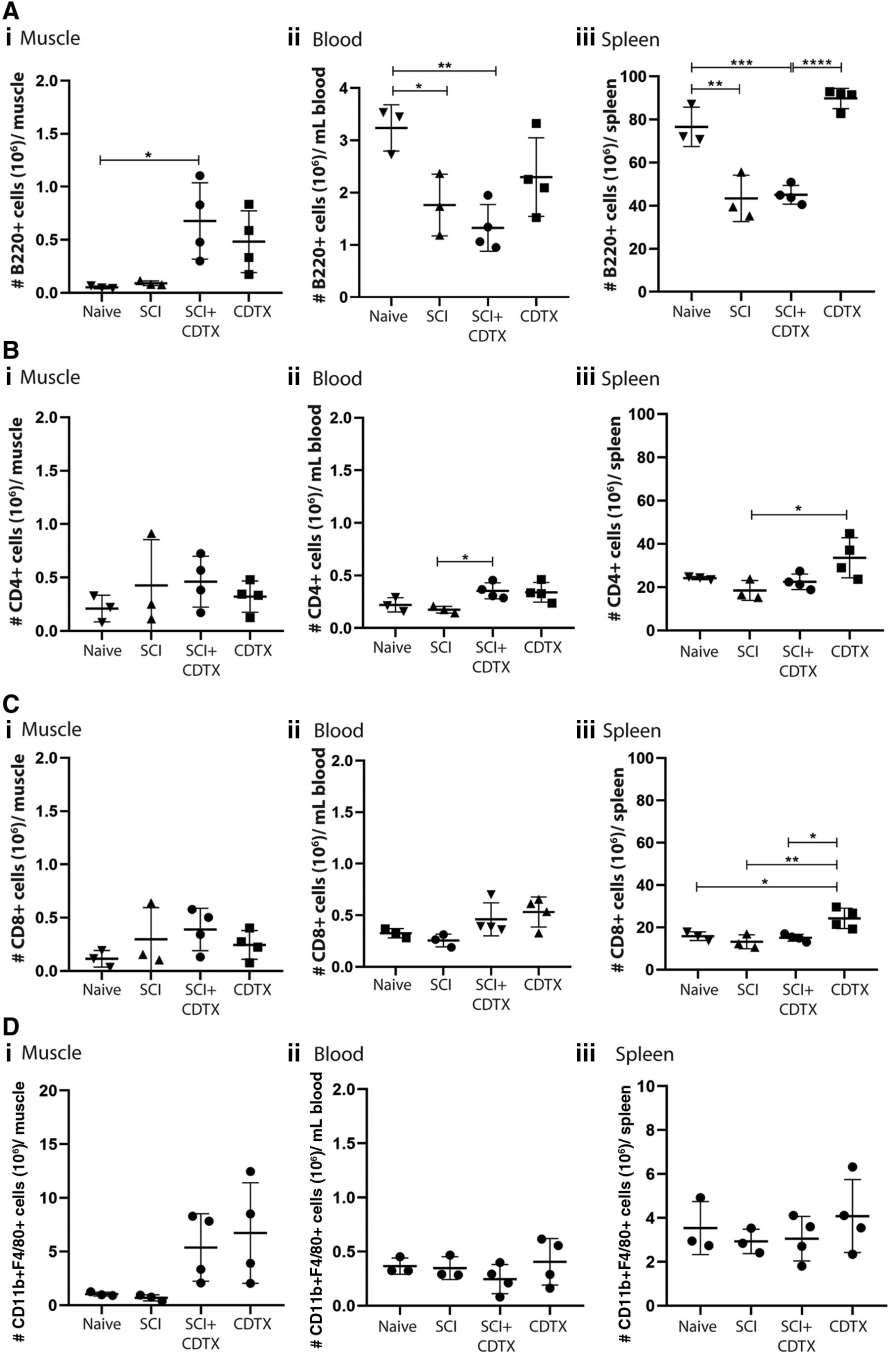
Lymphocyte populations present in tissues after spinal cord injury (SCI) and muscle damage. C57BL/6 mice underwent SCI surgery plus cardiotoxin (CDTX) injection, CDTX injection alone, SCI alone, or naïve as a control. Leukocytes were extracted from (i) muscle, (ii) blood, and (iii) spleen at four days post-surgery. (**A**) B lymphocyte numbers were identified via flow cytometry as (7AAD^-^ CD45^+^ CD3ɛ^-^B220^+^). (**B**) CD4 T lymphocytes were identified as 7AAD^-^ CD45^+^ CD3ɛ^+^ CD4^+^. (**C**) CD8 T lymphocytes were identified as 7AAD^-^ CD45^+^ CD3ɛ^+^ CD8^+^. (**D**) Monocyte/macrophages were identified as 7AAD^-^ CD45^+^ CD11b^+^ F4/80^+^. Each dot represents one mouse; results are presented as mean ± standard deviation, single experiment, one way analysis of variance with Tukey multiple comparisons test; *p < 0.5, **p < 0.01, ***p < 0.001, and ****p < 0.0001.

Flow cytometry showed an increase in CD45R/B220^+^ B lymphocytes in injured muscles in which NHO was developing (Fig. 1Ai). B lymphocyte numbers were similarly increased in CDTX-injected muscles of mice without SCI, suggesting that B lymphocytes are recruited to injured muscles regardless of the presence of a concomitant SCI. CD3ɛ^+^CD4^+^ (Fig. 1Bi) and CD3ɛ^+^CD8^+^ (Fig. 1Ci) T cells were also detected within muscle samples; however, their frequency did not change between groups. Overall, lymphocytes were lower in frequency (all <10^6^ cells/hamstring in the SCI+CDTX group) in muscles compared with innate immune cells such as monocyte/macrophages (5.8 × 10^6^ cells/hamstring in the SCI+CDTX group)(Fig. 1Di).

In the blood, SCI was associated with a significant decrease in the number of B lymphocytes compared with naïve control mice (Fig. 1Aii), and this effect was even more pronounced in the spleen (Fig. 1Aiii). This observation is consistent with reported lymphopenia subsequent to SCI at thoracic levels T1, T3, and/or T9.^39,43^ The SCI-induced lymphopenia seen here most profoundly affected B lymphocytes with no or only minimal impact on T lymphocyte and monocyte/macrophage frequencies in both the blood and the spleen (Fig. 1Bii-iii, Cii-iii, Dii-iii). No lymphopenia was observed in association with CDTX injection alone, highlighting the specific contribution of SCI in this phenomenon.

### NHO develops in *Rag1*^-/-^ mice similar to wild type control mice

**FIG. 2.**
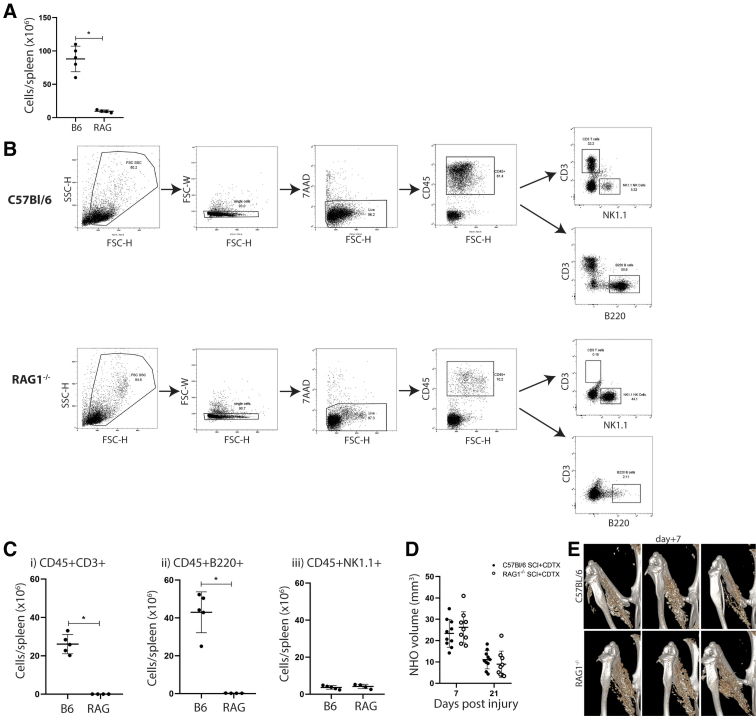
Mature T and B lymphocytes are not required for spinal cord injury-neurogenic heterotopic ossification (SCI-NHO). (**A**) Total spleenocyte counts from C57Bl/6 (B6) and *Rag1^-/-^* (RAG) mice 21 days after SCI and cardiotoxin (CDTX) muscle injury, *p = 0.0159. (**B**) Flow cytometry gating strategy used to identify splenic lymphocyte populations from either B6 or RAG mice post-SCI+CDTX. (**C**) Frequency of either (i) CD45^+^ CD3ɛ^+^ T cells, *p = 0.0159, (ii) CD45^+^ B220^+^ B cells, *p = 0.0159, and (iii) CD45^+^ NK1.1^+^ NK cells, confirming the absence of mature T and B cells in RAG mice 21 days post-SCI+CDTX. (**D**) Measurement of NHO bone volume by μCT in B6 or RAG mice at seven and 21 days post- SCI+CDTX. (**E**) Representative μCT images at seven days post-surgery in C57BL/6 and *Rag1^-/-^* mice. Each dot represents an individual mouse; data represented as mean ± standard deviation, two experiments, Mann Whitney *U* test.

We next used *Rag1*^-/-^ mice to investigate directly a possible contribution of mature T and B lymphocytes to NHO development. The total splenic cellularity of *Rag1*^-/-^ mice was significantly reduced compared with wild-type controls (Fig. 2A). Flow cytometry confirmed that *Rag1*^-/-^ mice lacked mature T and B lymphocytes compared with C57BL/6 control mice (Fig. 2B, Ci-ii).^37^ We also investigated natural killer (NK) cell frequency, which remained unaltered (Fig. 2Ciii). Both C57BL/6 and *Rag1*^-/-^ mice underwent SCI together with an intramuscular injection of CDTX. NHO development was monitored by μCT at both seven and 21 days post-surgery. Quantification of NHO volumes at both time points showed that absence of mature T and B lymphocytes did not impact NHO development. (Fig. 2D-E).

### Splenectomy does not affect NHO after SCI

**FIG. 3.**
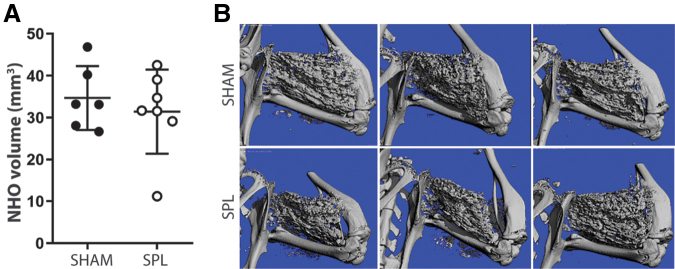
Splenectomy does not impact neurogenic heterotopic ossification (NHO) development. (**A**) C57BL/6 mice underwent either abdominal sham or splenectomy (SPL) surgery before SCI surgery and cardiotoxin-induced muscle injury. NHO development was measured at 10 days post-surgery. (**B**) Representative μCT images at 10 days post-surgery. Each dot represents an individual mouse; data represented as mean ± standard deviation, single experiment.

Lastly, considering that the spleen is the largest lymphatic organ and a proposed site of emergency monocytopoiesis,^44^ we examined whether surgical removal of the spleen influenced NHO development. μCT, performed at 10 days post-surgery, revealed no impact of splenectomy on ectopic bone formation within CDTX-injected muscles of SCI mice (Fig. 3A-B). These findings are in agreement with those observed in *Rag1*^-/-^ mice and also rule out a putative role of the splenic monocyte reservoir in myeloid-driven NHO development.^12,13,16^

## Discussion

While previous studies have largely focused on the contributions of innate immune cells such as macrophages and mast cells to the development of genetically driven HO,^19,20^ non-neurogenic trauma-acquired HO^19–22^ and NHO,^12,13,16,18^ in this study we establish that T and B lymphocyte populations make minimal or redundant contributions to experimentally-induced NHO after SCI.

We demonstrated that T and B lymphocytes are present at sites of muscle injury (day 4 post-surgery); however, they were lower in frequency compared with innate immune cells such as monocyte/macrophages. Similar to other murine models of SCI (T1, T3, or T9),^39,43^ we noted a reduction in CD45R/B220^+^ B lymphocytes in spleen as well as the blood after SCI. Interestingly, the frequency of B lymphocytes was increased in injured muscles of mice with SCI, with no or minimal changes in T lymphocyte frequencies. Nonetheless, we subsequently show that *Rag1*^-/-^ mice, which lack both mature T and B cell subsets, developed similar volumes of NHO to wild type cohorts. Because the spleen is a major reservoir of B and T lymphocytes, we undertook splenectomy before SCI surgery and muscle injury. Splenectomy, however, did not alter NHO development either thereby also ruling out a role for the splenic monocyte reservoir in this pathology.

Overall, we demonstrate that lymphocytes are present at relatively small frequencies within injured muscles and may still contribute to early inflammatory responses, similar to our recent study on the role of neutrophils^18^; however, the contribution of lymphocytes may be “substituted” by other immune cells present within injured muscles.

Interestingly, others have established a role for T lymphocytes in CDTX-induced muscle repair. Loss of CD8^+^ T cells resulted in impaired muscle regeneration and increased matrix deposition because of failed recruitment of macrophages to the injury site.^45^ Depletion of regulatory T cells has also been shown to impair muscle regeneration because of the prolonged presence of inflammatory cells in damaged muscle that subsequently impaired the regeneration process.^46^ However, we did not evaluate muscle repair in our model of SCI-NHO.

Our findings on the redundant role of lymphocytes in SCI-NHO are in stark contrast to those in HO induced by non-neurological trauma. Specifically, in a model of traumatic HO (skin burn injury + Achilles tenotomy), *Rag*1^-/-^ mice displayed significantly reduced HO formation.^25^ *Rag1*^-/-^ mice also displayed no alteration in osteoclast number or activation in this model, but had reduced expression of endochondral ossification markers such as *Sox9* and *Bglap* encoding osteocalcin.^25^ These differences highlight the complexities between different models of HO, where the underlying mechanisms driving traumatic HO (partial skin thickness burn injury + Achilles tenotomy) versus NHO subsequent to a severe injury of the CNS (complete spinal cord transection + muscle injury) may not overlap completely.^47^

Indeed, although macrophage-mediated inflammatory responses driving genetically acquired and trauma-induced HO seem to overlap,^12,19,21,48^ the permissive molecular events that enable these different forms of HO to develop are different. Specifically, HO in FOP are caused by gain-of-function dominant missense mutation of the *ACVR1* gene encoding a type 1 bone morphogenetic protein receptor,^48–50^ whereas NHO are in part driven by excessive adrenergic signaling as a consequence of a SCI.^51^ Which molecular events are permissive to HO development after severe burn injuries remain yet to be determined.

Lastly, there are important limitations of our study to consider because we did not determine the contribution of each lymphocyte population independently. In addition, while our mouse model of NHO recapitulates many features of NHO observed in patients with SCI,^12^ a limitation of our model is that it does not recapitulate the endochondral phase observed in human NHO. We have previously established that NHO forms in our mouse model via intramembranous ossification,^14,15^ which could also potentially explain our contrasting results with those in non-neurologic models of traumatic HO.

## Conclusion

We document that targeting the adaptive immune response may not be as beneficial as targeting innate immune responses via monocytes/macrophages, which we have already shown to be successful.^13,14,16^

## Abbreviations Used

ANOVA: analysis of variance
APC: allophycocyanin
CDTX: cardiotoxin
CNS: central nervous system
FCS: fetal calf serum
FITC: fluorescein isothiocyanate
FOP: fibrodysplasia ossificans progressiva
HO: heterotopic ossifications
HU: Hounsfield unit
IL: interleukin
IM: intramuscular
JAK: Janus kinase
μCT: micro computed tomography
NHO: neurogenic heterotopic ossifications
NK: natural killer
PBS: phosphate buffered saline
PE: phycoerythrin
PET-CT: positron emission tomography – computed tomography
RAG: *Rag1* ^-/-^
ROI: region of interest
SCI: spinal cord injury
SD: standard deviation
STAT: signal transducer and activator of transcription
TBI: traumatic brain injury
3D: three dimensional

## References

[1] Ohlmeier, M., Suero, E.M., Aach, M., Meindl, R., Schildhauer, T.A., and Citak, M. (2017). Muscle localization of heterotopic ossification following spinal cord injury. Spine J. 17, 1519–1522. 28456672 10.1016/j.spinee.2017.04.021

[2] Genet, F., Jourdan, C., Schnitzler, A., Lautridou, C., Guillemot, D., Judet, T., Poiraudeau, S., and Denormandie, P. (2011). Troublesome heterotopic ossification after central nervous system damage: a survey of 570 surgeries. PLoS One 6, e16632. 21304993 10.1371/journal.pone.0016632 PMC3031592

[3] Dizdar, D., Tiftik, T., Kara, M., Tunc, H., Ersoz, M., and Akkus, S. (2013). Risk factors for developing heterotopic ossification in patients with traumatic brain injury. Brain Inj. 27, 807–811. 23730889 10.3109/02699052.2013.775490

[4] Reznik, J.E., Biros, E., Marshall, R., Jelbart, M., Milanese, S., Gordon, S., and Galea, M.P. (2014). Prevalence and risk-factors of neurogenic heterotopic ossification in traumatic spinal cord and traumatic brain injured patients admitted to specialised units in Australia. J. Musculoskelet. Neuronal Interact. 14, 19–28. 24583537

[5] Brady, R.D., Shultz, S.R., McDonald, S.J., and O'Brien, T.J. (2018). Neurological heterotopic ossification: current understanding and future directions. Bone 109, 35–42. 28526267 10.1016/j.bone.2017.05.015

[6] Forsberg, J.A., Pepek, J.M., Wagner, S., Wilson, K., Flint, J., Andersen, R.C., Tadaki, D., Gage, F.A., Stojadinovic, A., and Elster, E.A. (2009). Heterotopic ossification in high-energy wartime extremity injuries: prevalence and risk factors. J. Bone Joint Surg. Am. 91, 1084–1091. 19411456 10.2106/JBJS.H.00792

[7] Vanden Bossche, L,. and Vanderstraeten, G. (2005). Heterotopic ossification: a review. J. Rehabil. Med. 37, 129–136. 16040468 10.1080/16501970510027628

[8] Salga, M., Jourdan, C., Durand, M.C., Hangard, C., Denormandie, P., Carlier, R.Y., and Genêt, F. (2015). Sciatic nerve compression by neurogenic heterotopic ossification: use of CT to determine surgical indications. Skeletal Radiol. 44, 233–240. 25218150 10.1007/s00256-014-2003-6

[9] Genet, F., Chehensse, C., Jourdan, C., Lautridou, C., Denormandie, P., and Schnitzler, A. (2012). Impact of the operative delay and the degree of neurologic sequelae on recurrence of excised heterotopic ossification in patients with traumatic brain injury. J. Head Trauma Rehabil. 27, 443–448. 22495100 10.1097/HTR.0b013e31822b54ba

[10] Genet, F., Marmorat, J.L., Lautridou, C., Schnitzler, A., Mailhan, L., and Denormandie, P. (2009). Impact of late surgical intervention on heterotopic ossification of the hip after traumatic neurological injury. J. Bone Joint Surg. Br. 91, 1493–1498. 19880896 10.1302/0301-620X.91B11.22305

[11] Genêt, F., Minooee, K., Jourdan, C., Ruet, A., Denormandie, P., and Schnitzler, A. (2015). Troublesome heterotopic ossification and stroke: features and risk factors. A case control study. Brain Inj. 29, 866–871. 25915823 10.3109/02699052.2015.1005133

[12] Genêt, F., Kulina, I., Vaquette, C., Torossian, F., Millard, S., Pettit, A.R., Sims, N.A., Anginot, A., Guerton, B., Winkler, I.G., Barbier, V., Lataillade, J.J., Le Bousse-Kerdilès, M.C., Hutmacher, D.W., and Levesque, J.P. (2015). Neurological heterotopic ossification following spinal cord injury is triggered by macrophage-mediated inflammation in muscle. J. Pathol. 236, 229–240. 25712044 10.1002/path.4519

[13] Alexander, K.A., Tseng, H.W., Fleming, W., Jose, B., Salga, M., Kulina, I., Millard, S.M., Pettit, A.R., Genêt, F., and Levesque, J.P. (2019). Inhibition of JAK1/2 tyrosine kinases reduces neurogenic heterotopic ossification after spinal cord injury. Front. Immunol. 10, 377. 30899259 10.3389/fimmu.2019.00377 PMC6417366

[14] Tseng, H.W., Kulina, I., Girard, D., Gueguen, J., Vaquette, C., Salga, M., Fleming, W., Jose, B., Millard, S.M., Pettit, A.R., Schroder, K., Thomas, G., Wheeler, L., Genêt, F., Banzet, S., Alexander, K.A., and Lévesque, J.P. (2021). Interleukin-1 is overexpressed in injured muscles following spinal cord injury and promotes neurogenic heterotopic ossification. J. Bone Miner. Res. Online ahead of print. 10.1002/jbmr.4482 34841579

[15] Tseng HW, G.D., Alexander KA, Millard SM, Torossian, F, Anginot A, Fleming W, Gueguen J, Goriot ME, Clay D, Jose B, Nowlan B, Pettit AR, Salga M, Genêt F, Le Bousse-Kerdilès MC, Banzet S, Levesque JP (2022). Spinal cord injury reprograms muscle fibro-adipogenic progenitors to form heterotopic bones within muscles. Bone Res. [Epub ahead of print; DOI: 10.1038/s41413-022-00188-y]. PMC8881504 35217633

[16] Torossian, F., Guerton, B., Anginot, A., Alexander, K.A., Desterke, C., Soave, S., Tseng, H.W., Arouche, N., Boutin, L., Kulina, I., Salga, M., Jose, B., Pettit, A.R., Clay, D., Rochet, N., Vlachos, E., Genet, G., Debaud, C., Denormandie, P., Genet, F., Sims, N.A., Banzet, S., Levesque, J.P., Lataillade, J.J., and Le Bousse-Kerdilès, M.C. (2017). Macrophage-derived oncostatin M contributes to human and mouse neurogenic heterotopic ossifications. JCI Insight 2, e96034. 10.1172/jci.insight.96034 PMC5752299 29093266

[17] Bisht, K., McGirr, C., Lee, S.Y., Tseng, H.W., Fleming, W., Alexander, K.A., Matsumoto, T., Barbier, V., Sims, N.A., Müller-Newen, G., Winkler, I.G., Bonig, H., and Lévesque, J.P. (2021). Oncostatin M regulates hematopoietic stem cell (HSC) niches in the bone marrow to restrict HSC mobilization. Leukemia. Online ahead of print. 10.1038/s41375-021-01413-z 34518644

[18] Tseng, H.W., Kulina, I., Salga, M., Fleming, W., Vaquette, C., Genêt, F., Levesque, J.P., and Alexander, K.A. (2020). Neurogenic heterotopic ossifications develop independently of granulocyte-colony stimulating factor and neutrophils. J. Bone Miner. Res. 35, 2242-–2251. 32568412 10.1002/jbmr.4118

[19] Convente, M.R., Chakkalakal, S.A., Yang, E., Caron, R.J., Zhang, D., Kambayashi, T., Kaplan, F.S., and Shore, E.M. (2018). Depletion of mast cells and macrophages impairs heterotopic ossification in an Acvr1(R206H) mouse model of fibrodysplasia ossificans progressiva. J. Bone Miner. Res. 33, 269–282. 28986986 10.1002/jbmr.3304 PMC7737844

[20] Kan, L., Liu, Y., McGuire, T.L., Berger, D.M., Awatramani, R.B., Dymecki, S.M., and Kessler, J.A. (2009). Dysregulation of local stem/progenitor cells as a common cellular mechanism for heterotopic ossification. Stem Cells 27, 150–156. 18832590 10.1634/stemcells.2008-0576 PMC2752983

[21] Sorkin, M., Huber, A.K., Hwang, C., Carson, W.F.t., Menon, R., Li, J., Vasquez, K., Pagani, C., Patel, N., Li, S., Visser, N.D., Niknafs, Y., Loder, S., Scola, M., Nycz, D., Gallagher, K., McCauley, L.K., Xu, J., James, A.W., Agarwal, S., Kunkel, S., Mishina, Y., and Levi, B. (2020). Regulation of heterotopic ossification by monocytes in a mouse model of aberrant wound healing. Nat. Commun. 11, 722. 32024825 10.1038/s41467-019-14172-4 PMC7002453

[22] Kraft, C.T., Agarwal, S., Ranganathan, K., Wong, V.W., Loder, S., Li, J., Delano, M.J., and Levi, B. (2016). Trauma-induced heterotopic bone formation and the role of the immune system: a review. J. Trauma Acute Care Surg. 80, 156–165. 26491794 10.1097/TA.0000000000000883 PMC4688132

[23] Kaplan, F., Shore, E., Gupta, R., Billings, P., Glaser, D., Pignolo, R., Graf, D., and Kamoun, M. (2005). Immunological features of fibrodysplasia ossificans progressiva and the dysregulated BMP4 pathway. Clinic Rev. Bone Miner. Metab. 3, 189–193.

[24] Gannon, F.H., Valentine, B.A., Shore, E.M., Zasloff, M.A., and Kaplan, F.S. (1998). Acute lymphocytic infiltration in an extremely early lesion of fibrodysplasia ossificans progressiva. Clin. Orthop. Relat. Res. 346:19–25. 9577406

[25] Ranganathan, K., Agarwal, S., Cholok, D., Loder, S., Li, J., Sung Hsieh, H.H., Wang, S.C., Buchman, S.R., and Levi, B. (2016). The role of the adaptive immune system in burn-induced heterotopic ossification and mesenchymal cell osteogenic differentiation. J. Surg. Res. 206, 53–61. 27916375 10.1016/j.jss.2016.04.040 PMC5532013

[26] Forsberg, J.A., Potter, B.K., Polfer, E.M., Safford, S.D, and Elster, E.A. (2014). Do inflammatory markers portend heterotopic ossification and wound failure in combat wounds? Clin. Orthop. Relat. Res. 472, 2845–2854. 24879568 10.1007/s11999-014-3694-7 PMC4117913

[27] Hoff, P., Rakow, A., Gaber, T., Hahne, M., Sentürk, U., Strehl, C., Fangradt, M., Schmidt-Bleek, K., Huscher, D., Winkler, T., Matziolis, D., Matziolis, G., Badakhshi, H., Burmester, G.R., Duda, G.N., Perka, C., and Buttgereit, F. (2013). Preoperative irradiation for the prevention of heterotopic ossification induces local inflammation in humans. Bone 55, 93–101. 23571050 10.1016/j.bone.2013.03.020

[28] Terauchi, M., Li, J.Y., Bedi, B., Baek, K.H., Tawfeek, H., Galley, S., Gilbert, L., Nanes, M.S., Zayzafoon, M., Guldberg, R., Lamar, D.L., Singer, M.A., Lane, T.F., Kronenberg, H.M., Weitzmann, M.N., and Pacifici, R. (2009). T lymphocytes amplify the anabolic activity of parathyroid hormone through Wnt10b signaling. Cell Metab. 10, 229–240. 19723499 10.1016/j.cmet.2009.07.010 PMC2751855

[29] Kim, Y.G., Lee, C.K., Nah, S.S., Mun, S.H., Yoo, B., and Moon, H.B. (2007). Human CD4+CD25+ regulatory T cells inhibit the differentiation of osteoclasts from peripheral blood mononuclear cells. Biochem. Biophys. Res. Commun. 357, 1046–1052. 17462597 10.1016/j.bbrc.2007.04.042

[30] Buchwald, Z.S., Kiesel, J.R., DiPaolo, R., Pagadala, M.S., and Aurora, R. (2012). Osteoclast activated FoxP3+ CD8+ T-cells suppress bone resorption in vitro. PLoS One 7, e38199. 22701612 10.1371/journal.pone.0038199 PMC3368916

[31] Weitzmann, M.N., Cenci, S., Haug, J., Brown, C., DiPersio, J., and Pacifici, R. (2000). B lymphocytes inhibit human osteoclastogenesis by secretion of TGFbeta. J. Cell. Biochem. 78, 318–324. 10842325 10.1002/(sici)1097-4644(20000801)78:2<318::aid-jcb13>3.0.co;2-n

[32] Kong, Y.Y., Feige, U., Sarosi, I., Bolon, B., Tafuri, A., Morony, S., Capparelli, C., Li, J., Elliott, R., McCabe, S., Wong, T., Campagnuolo, G., Moran, E., Bogoch, E.R., Van, G., Nguyen, L.T., Ohashi, P.S., Lacey, D.L., Fish, E., Boyle, W.J., and Penninger, J.M. (1999). Activated T cells regulate bone loss and joint destruction in adjuvant arthritis through osteoprotegerin ligand. Nature 402, 304–309. 10580503 10.1038/46303

[33] Horwood, N.J., Kartsogiannis, V., Quinn, J.M., Romas, E., Martin, T.J., and Gillespie, M.T. (1999). Activated T lymphocytes support osteoclast formation in vitro. Biochem. Biophys. Res. Commun. 265, 144–150. 10548505 10.1006/bbrc.1999.1623

[34] Toben, D., Schroeder, I., El Khassawna, T., Mehta, M., Hoffmann, J.E., Frisch, J.T., Schell, H., Lienau, J., Serra, A., Radbruch, A., and Duda, G.N. (2011). Fracture healing is accelerated in the absence of the adaptive immune system. J. Bone Miner. Res. 26, 113–124. 20641004 10.1002/jbmr.185

[35] Nam, D., Mau, E., Wang, Y., Wright, D., Silkstone, D., Whetstone, H., Whyne, C., and Alman, B. (2012). T-lymphocytes enable osteoblast maturation via IL-17F during the early phase of fracture repair. PLoS One 7, e40044. 22768215 10.1371/journal.pone.0040044 PMC3386936

[36] Mombaerts, P., Iacomini, J., Johnson, R.S., Herrup, K., Tonegawa, S., and Papaioannou, V.E. (1992). RAG-1-deficient mice have no mature B and T lymphocytes. Cell 68, 869–877. 1547488 10.1016/0092-8674(92)90030-g

[37] Raggatt, L.J., Alexander, K.A., Kaur, S., Wu, A.C., MacDonald, K.P., and Pettit, A.R. (2013). Absence of B cells does not compromise intramembranous bone formation during healing in a tibial injury model. Am. J. Pathol. 182, 1501–1508. 23499466 10.1016/j.ajpath.2013.01.046

[38] Meisel, C., Schwab, J.M., Prass, K., Meisel, A., and Dirnagl, U. (2005). Central nervous system injury-induced immune deficiency syndrome. Nat. Rev. Neurosci. 6, 775–786. 16163382 10.1038/nrn1765

[39] Zhang, Y., Guan, Z., Reader, B., Shawler, T., Mandrekar-Colucci, S., Huang, K., Weil, Z., Bratasz, A., Wells, J., Powell, N.D., Sheridan, J.F., Whitacre, C.C., Rabchevsky, A.G., Nash, M.S., and Popovich, P.G. (2013). Autonomic dysreflexia causes chronic immune suppression after spinal cord injury. J. Neurosci. 33, 12970–12981. 23926252 10.1523/JNEUROSCI.1974-13.2013 PMC3735880

[40] Jogia, T., Lübstorf, T., Jacobson, E., Scriven, E., Atresh, S., Nguyen, Q.H., Liebscher, T., Schwab, J.M., Kopp, M.A., Walsham, J., Campbell, K.E., and Ruitenberg, M.J. (2021). Prognostic value of early leukocyte fluctuations for recovery from traumatic spinal cord injury. Clin. Transl. Med. 11, e272. 33463065 10.1002/ctm2.272 PMC7805435

[41] Reeves, J.P., Reeves, P.A., and Chin, L.T. (2001). Survival surgery: removal of the spleen or thymus. Curr. Protoc. Immunol. Chapter 1, Unit 1.10. 10.1002/0471142735.im0110s02 18432668

[42] Barbier, V., Winkler, I.G., and Levesque, J.P. (2012). Mobilization of hematopoietic stem cells by depleting bone marrow macrophages. Methods Mol. Biol. 904, 117–138. 22890928 10.1007/978-1-61779-943-3_11

[43] Prüss, H., Tedeschi, A., Thiriot, A., Lynch, L., Loughhead, S.M., Stutte, S., Mazo, I.B., Kopp, M.A., Brommer, B., Blex, C., Geurtz, L.-C., Liebscher, T., Niedeggen, A., Dirnagl, U., Bradke, F., Volz, M.S., DeVivo, M.J., Chen, Y., von Andrian, U.H., and Schwab, J.M. (2017). Spinal cord injury-induced immunodeficiency is mediated by a sympathetic-neuroendocrine adrenal reflex. Nat. Neurosci. 20, 1549–1559. 28920935 10.1038/nn.4643

[44] Swirski, F.K., Nahrendorf, M., Etzrodt, M., Wildgruber, M., Cortez-Retamozo, V., Panizzi, P., Figueiredo, J.L., Kohler, R.H., Chudnovskiy, A., Waterman, P., Aikawa, E., Mempel, T.R., Libby, P., Weissleder, R., and Pittet, M.J. (2009). Identification of splenic reservoir monocytes and their deployment to inflammatory sites. Science 325, 612–616. 19644120 10.1126/science.1175202 PMC2803111

[45] Zhang, J., Xiao, Z., Qu, C., Cui, W., Wang, X., and Du, J. (2014). CD8 T cells are involved in skeletal muscle regeneration through facilitating MCP-1 secretion and Gr1(high) macrophage infiltration. J. Immunol. 193, 5149–5160. 25339660 10.4049/jimmunol.1303486

[46] Burzyn, D., Kuswanto, W., Kolodin, D., Shadrach, J.L., Cerletti, M., Jang, Y., Sefik, E., Tan, T.G., Wagers, A.J., Benoist, C., and Mathis, D. (2013). A special population of regulatory T cells potentiates muscle repair. Cell 155, 1282–1295. 24315098 10.1016/j.cell.2013.10.054 PMC3894749

[47] Levesque, J.P., Sims, N.A., Pettit, A.R., Alexander, K.A., Tseng, H.W., Torossian, F., Genêt, F., Lataillade, J.J., and Le Bousse-Kerdilès, M.C. (2018). Macrophages driving heterotopic ossification: convergence of genetically-driven and trauma-driven mechanisms. J. Bone Miner. Res. 33, 365–366. 29178621 10.1002/jbmr.3346

[48] Shore, E.M., Xu, M., Feldman, G.J., Fenstermacher, D.A., Cho, T.J., Choi, I.H., Connor, J.M., Delai, P., Glaser, D.L., LeMerrer, M., Morhart, R., Rogers, J.G., Smith, R., Triffitt, J.T., Urtizberea, J.A., Zasloff, M., Brown, M.A., and Kaplan, F.S. (2006). A recurrent mutation in the BMP type I receptor ACVR1 causes inherited and sporadic fibrodysplasia ossificans progressiva. Nat. Genet. 38, 525–527. 16642017 10.1038/ng1783

[49] Hatsell, S.J., Idone, V., Wolken, D.M., Huang, L., Kim, H.J., Wang, L., Wen, X., Nannuru, K.C., Jimenez, J., Xie, L., Das, N., Makhoul, G., Chernomorsky, R., D'Ambrosio, D., Corpina, R.A., Schoenherr, C.J., Feeley, K., Yu, P.B., Yancopoulos, G.D., Murphy, A.J., and Economides, A.N. (2015). ACVR1R206H receptor mutation causes fibrodysplasia ossificans progressiva by imparting responsiveness to activin A. Sci .Transl. Med. 7, 303ra137. 10.1126/scitranslmed.aac4358 PMC6164166 26333933

[50] Hildebrand, L., Stange, K., Deichsel, A., Gossen, M., and Seemann, P. (2017). The Fibrodysplasia Ossificans Progressiva (FOP) mutation p.R206H in ACVR1 confers an altered ligand response. Cell. Signal. 29, 23–30. 27713089 10.1016/j.cellsig.2016.10.001

[51] Debaud, C., Tseng, H.W., Chedik, M., Kulina, I., Genêt, F., Ruitenberg, M.J., and Levesque, J.P. (2021). Local and systemic factors drive ectopic osteogenesis in regenerating muscles of spinal-cord-injured mice in a lesion-level-dependent manner. J. Neurotrauma 38, 2162–2175. 33913747 10.1089/neu.2021.0058

